# Efficacy of single-site radiotherapy plus PD-1 inhibitors vs PD-1 inhibitors for oligometastatic non-small cell lung cancer

**DOI:** 10.1007/s00432-021-03849-3

**Published:** 2021-11-23

**Authors:** Peiliang Wang, Tianwen Yin, Kaikai Zhao, Jinming Yu, Feifei Teng

**Affiliations:** 1 Department of Radiation Oncology, Shandong Cancer Hospital and Institute, Cheello College of Medicine, Shandong University, Jiyan Road 440, Jinan, 250117 Shandong Province People’s Republic of China; 2 Department of Radiation Oncology, Yantai Affiliated Hospital of Binzhou Medical University, Yantai, Shandong People’s Republic of China; 3 Department of Radiation Oncology, Shandong Cancer Hospital and Institute, Shandong First Medical University and Shandong Academy of Medical Sciences, Jinan, Shandong People’s Republic of China

**Keywords:** Oligometastatic, PD-1, Immune checkpoint inhibitors, Radiotherapy, NSCLC

## Abstract

**Purpose:**

Growing numbers of clinical trials test the efficacy of radiotherapy (RT) plus immune checkpoint inhibitors (ICIs), but the number of irradiated sites is not uniform. We aimed to evaluate the efficacy of single-site RT plus immunotherapy in oligometastatic non-small cell lung cancer (NSCLC) with smaller disease burdens and low tumor heterogeneity.

**Methods:**

We retrospectively identified oligometastatic NSCLC (< 4 metastatic sites) patients treated with PD-1 pathway inhibitors with or without RT to a single lesion in our institution between 2018 and 2020. The primary endpoints were the best objective response rate (ORR) and progression-free survival (PFS).

**Results:**

Of the 152 patients enrolled, 93 and 59 were identified as the ICI alone group and the ICI plus RT group, respectively. The addition of RT to ICI therapy significantly increased the best ORR from 31.2% to 50.8% (*p* = 0.015). The out-of-field (abscopal effect) response rate could reach 41.3% (95%CI 26.5%–56.1%) in the ICI plus RT group. Median PFS was 8.9 months (95%CI 4.7–13.1 months) with ICI alone versus 13.8 months (95%CI 9.5–18.1 months) with ICI plus radiotherapy (hazard ratio [HR] 0.556; *p* = 0.035). In an exploratory subgroup analysis of PFS, the addition of RT brought greater benefits in patients aged < 65 years (*p* = 0.016), patients with ECOG PS = 0 (*p* = 0.048), and patients with 1–2 metastatic sites (*p* = 0.024). No unexpected adverse events or significantly increased toxicities were observed in the experimental arm.

**Conclusion:**

Single-site RT plus anti-PD-1 inhibitors significantly increased systemic responses and improved survival outcomes in oligometastatic NSCLC patients.

**Supplementary Information:**

The online version contains supplementary material available at 10.1007/s00432-021-03849-3.

## Background

The introduction of immunotherapy has transformed the treatment paradigm for advanced non-small cell lung cancer (NSCLC). Immunotherapy mainly refers to checkpoint inhibitors, such as PD-1, PD-L1, and CTLA-4. Chemotherapy combined with PD-1/PD-L1 inhibitors immunotherapy or immunotherapy alone has been approved as the standard first-line treatment for advanced NSCLC based on the results of the KEYNOTE (010, 024, 042) (Herbst et al. 2016; Mok et al. 2019; Reck et al. 2016). Unfortunately, only approximately 20% of unselected NSCLC patients could benefit from immunotherapy, spurring efforts to explore combination strategies (Borghaei et al. 2015; Gandhi et al. 2018).

Radiotherapy (RT) increases the expression of tumor-associated antigens and causes tumor cell immunogenic death, which promotes migration of T lymphocytes to tumor sites, thereby enhancing the local antitumor effects (Deng et al. 2014; Formenti and Demaria 2013; Verbrugge et al. 2012). Furthermore, RT can cause the decrease or regression of tumor outside the irradiation field. This phenomenon called the abscopal effect is because local RT causes a systemic immune response (Formenti et al. 2018; Khalife et al. 2019; Sezen et al. 2021; Theelen et al. 2020; Zhuang 2020).

However, data on the efficacy of the anti-PD-1 treatment with RT among metastatic NSCLC patients generally do not show better results than those among patients who have received immune checkpoint.

Inhibitors (ICI) alone (Samuel et al. 2020; Theelen et al. 2019). The reason for the inconsistent outcomes may be the single-site irradiation in these studies. Given the larger tumor burden and non-equal immunogenicity in metastatic NSCLC, irradiating only a single lesion in patients with multiple metastases might not be sufficient to induce systemic responses (Brooks and Chang 2019).

In metastatic NSCLC patients, approximately 25–50% of patients presented with oligometastatic disease (Parikh et al., 2014). (Bauml et al. 2019) conducted a single-arm phase II trial specifically focusing on oligometastatic NSCLC patients treated with local ablative therapies at all sites, plus pembrolizumab. The results with a 19.1-month median PFS-P (from the start date of pembrolizumab use) were significantly better than the historical control, with a PFS of 6.6 months. Despite this success, the irradiation of a single lesion continues to be the cornerstone of current strategies designed to test the efficacy of RT in combination with immunotherapy. Moreover, no prospective studies have been conducted on this trial design for oligometastatic NSCLC.

Therefore, we conducted a retrospective study of immunotherapy with or without irradiation of a single lesion for oligometastatic NSCLC. This study evaluated whether single-site RT was sufficient to enhance the systemic response of immunotherapy.

## Materials and methods

### Patient selection

**Fig. 1:**
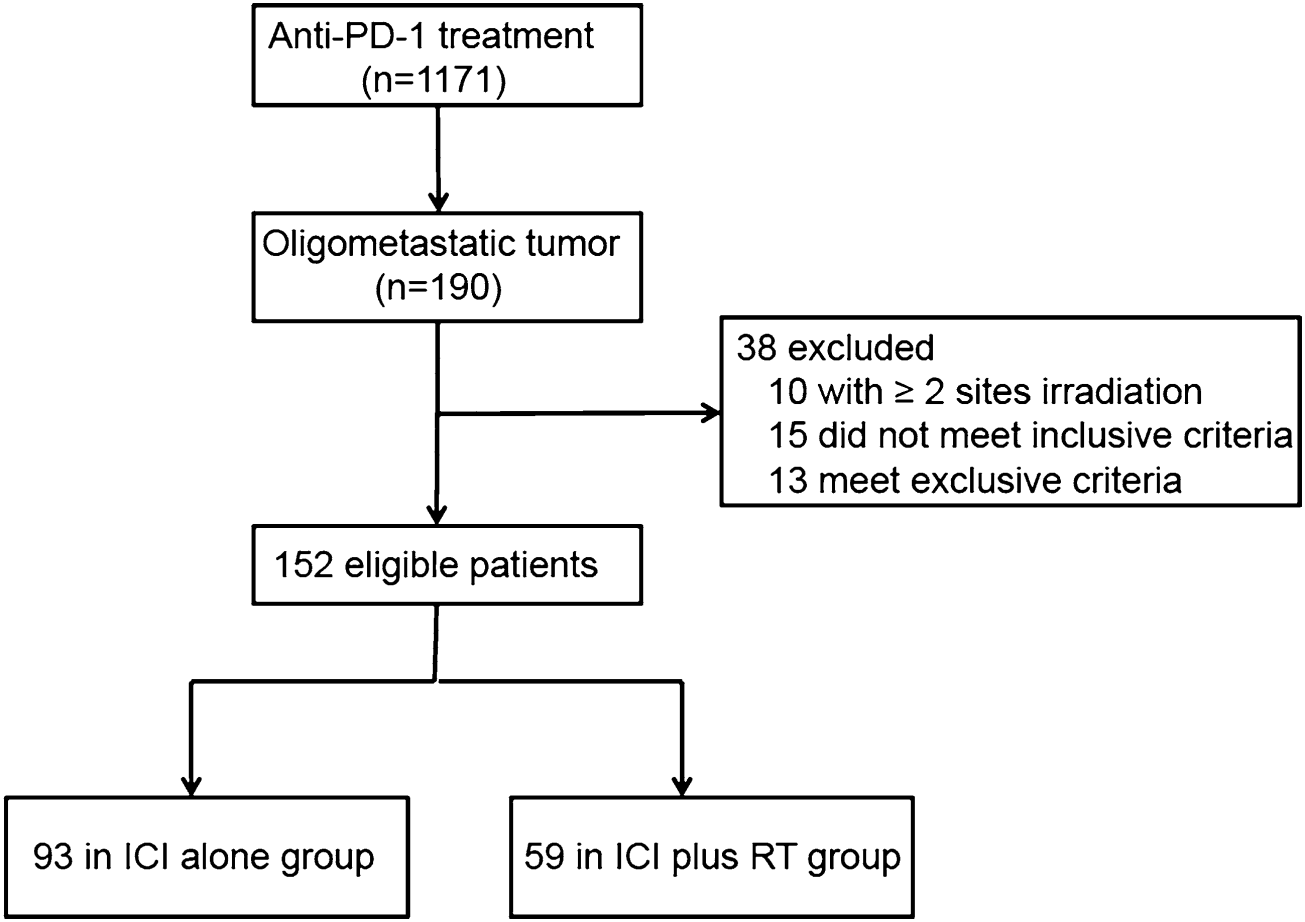
The flow chart of patient selection

Management of oligometastatic disease in patients with limited metastases is supported by relatively high-level evidence. In our institution, local therapies are recommended in combination with systemic therapy in well-selected patients. Gains in survival due to the ICIs therapy have further inspired research into the oligometastatic paradigm. We retrospectively reviewed the data of patients with oligometastatic NSCLC (defined as having < 4 metastases) (Bauml et al. 2019) treated in our institution (2018–2020) with immunotherapy (PD-1 checkpoint inhibitors) combined with or without RT (Fig. 1). The RT was limited to single-site irradiation in this treatment phase. Other key eligibility criteria included: (1) At least 1 separate lesion was required, which was measurable according to the Response Evaluation Criteria in Solid Tumors version 1.1; (2) ≥ 2 cycles of anti-PD-1 treatment; and (3) no epidermal growth factor receptor (EGFR) and/or anaplastic lymphoma kinase (ALK) targetable mutations. Patients were ineligible if they had (1) prior treatment with immunotherapy and (2) no complete clinical and follow-up data. Patients were divided into two groups based on whether they received RT or not: ICI alone and ICI plus RT groups. The collected data included baseline demographics, ECOG performance status, prior systemic treatment, immunotherapy regimens and RT details, treatment-related toxicities, and follow-up data.

The study was conducted in accordance with the Declaration of Helsinki (as revised in 2013). The study was approved by the Research Ethics Board of Shandong Cancer Hospital, and individual consent was waived owing to its retrospective nature. The authors are accountable for all aspects of the work in ensuring that questions relating to the accuracy or integrity of any part of the work are appropriately investigated and resolved.

### Treatment and outcomes

Patients received one of the following anti-PD-1 agents every two or three weeks with or without chemotherapy: sintilimab (Innovent Biologics, China), toripalimab (Shanghai Merck & Co.), camrelizumab (Jiangsu Hengrui Medicine, China), nivolumab (Bristol-Myers Squibb, USA), or pembrolizumab (Merck & Co., USA) (Supplementary Table 1). In the anti-PD-1 plus RT group, the radiation sites included primary tumors and metastatic lesions. Tumor response was assessed using radiographic imaging by the investigators according to Response Evaluation Criteria in Solid Tumors version 1.1 (RECIST 1.1), and adverse events (AEs) were evaluated using the Common Terminology Criteria for Adverse Events (CTCAE) version 4.03, with causality to treatment recorded. In particular, the response of unirradiated lesions (out-of-field) was also evaluated in the anti-PD-1 plus RT group.

The primary endpoints were the best objective response rate (ORR) and progression-free survival (PFS). Secondary endpoints included safety and disease control rates. PFS was defined as the time between the commencement of anti-PD-1 treatment to the date of progression or death, whichever occurred first.

### Statistical analyses

Baseline characteristics and quality-of-life measures were summarized by descriptive statistics and compared using χ2 contingency analyses. The Kaplan–Meier method and log-rank test were used to evaluate PFS. In the subgroup analyses, the effect on PFS of the addition of RT to immunotherapy was assessed among the subgroups using Cox proportional hazard models presented in a forest plot. Statistical significance was set at *P* < 0.05. Analyses were conducted using the Statistical Package for the Social Sciences software package, version 23.0 (SPSS Inc., Chicago, IL, USA) and GraphPad Prism, version 7.00 for Windows (GraphPad Software).

## Results

### Patient characteristics and disposition

Between July 2018 and March 2020, 152 eligible patients were retrospectively identified and assigned to the ICI alone group (*n* = 93) and the ICI plus RT group (*n* = 59). Figure 1 shows the selection of patients and patient characteristics are summarized in Table 1. The median age of these patients was 62 years (range, 34–81 years), and 128 (84%) were male. There were 39 (42.0%) patients in the ICI alone group and 21 (35.6%) patients in the ICI plus RT group receiving PD-1 inhibitor as first-line therapy. The PD-L1 status was collected in 50 patients. Patient demographics, including age, gender, smoking status, ECOG PS, histology, metastatic timing, number of metastases, previous chemotherapy, and systemic treatment options, were well balanced between the two groups (Table 1).

**Table 1:** Patient baseline clinical and treatment characteristics

Demographic or Characteristic	PD-1 (*n* = 93)	PD-1 plus RT (*n* = 59)	*P* value
*Age*			0.474
< 65	59	34	
≥ 65	34	25	
*Gender*			0.442
Male	80	48	
Female	13	11	
*Smoking, pack-years*			
< 10	35	28	0.231
≥ 10	58	31	
*ECOG PS*			
0	44	24	0.423
1–2	49	35	
*Histology*			
Adenocarcinoma	55	42	0.132
Squamous	38	17	
*Metastatic timing*			
Synchronous	55	28	0.159
Metachronous	38	31	
*Number of metastases*			0.241
1	33	28	
2	29	20	
3	23	8	
4	8	3	
*Lines of previous chemotherapy*			0.436
0	39	21	
1–3	54	38	
*PD-L1 status*			0.573
Negative	10	4	
Positive (≥ 1%)	20	16	
Unknown	63	39	
*Systemic treatment options*			0.138
Anti-PD-1 monotherapy	27	24	
Anti-PD-1 and chemotherapy	66	35	
*Irradiated tumor site*			
Lung, primary tumor	–	12	–
Lung/Pleural, metastasis	–	5	–
Brain	–	26	–
Bone	–	11	–
Adrenal	–	1	–
Liver	–	1	–
Lymph node(s)	–	3	–

*Abbreviations* *ECOG PS* Eastern Cooperative Oncology Group performance status, *PD-L1* programmed death ligand 1

### Efficacy

**Fig. 2:**
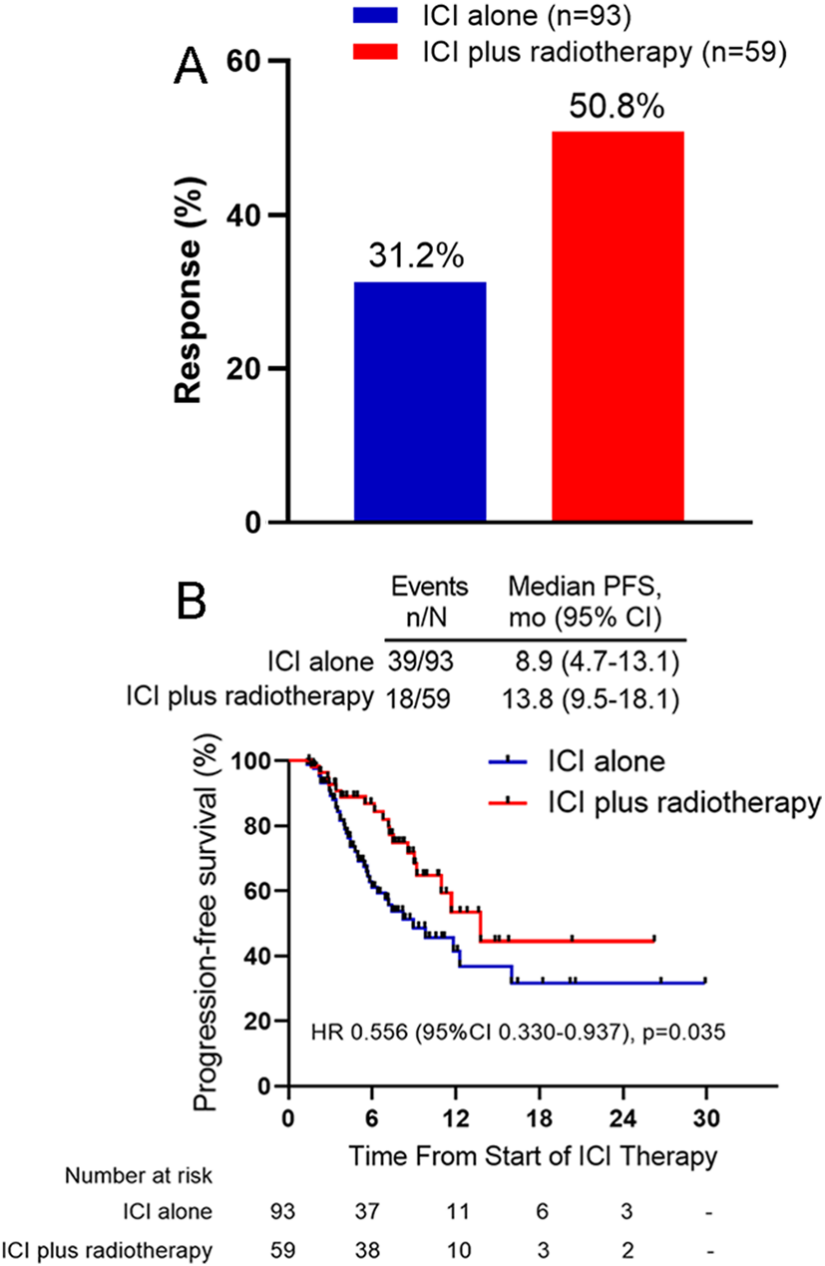
Best objective response rate (ORR) in patients for the ICI alone treatment versus ICI plus RT treatment comparison (**A**). Kaplan–Meier estimates of progression-free survival (PFS) in patients for the ICI alone versus ICI plus RT treatment comparison (**B**). *ICI *Immune checkpoint inhibitor, *mo *months, *HR *Hazard ratio, *CI *Confidence interval

At the cutoff date of February 2021, the median follow-up time was 8.1 months (range, 1.3–29.9 months). In the ICI alone group, no complete response was observed, 29 (31.2%) patients had confirmed partial response, and 52 (55.9%) patients had stable disease (Table 2). In the ICI plus RT group, 2 (3.4%) patients had confirmed complete response, 28 (47.5%) patients achieved partial response, and 25 (42.4%) had stable disease (Table 2). Best ORR was significantly higher with ICI plus RT compared with ICI alone (50.8% vs. 31.2%; odds ratio [OR] 2.28, 95% CI 1.17–4.48; *p* = 0.015) (Table 2 and Fig. 2A). In the out-of-field evaluable population (*n* = 46) of the ICI plus RT group, the out-of-field ORR was 41.3% (95% CI, 26.5–56.1%), which is higher than an ORR of 31.2% in the ICI alone group (*p* = 0.238) (Table2). Kaplan–Meier analysis indicated a significantly better PFS in the ICI plus RT group compared with the ICI alone group (median PFS, 13.8 vs. 8.9 months; HR, 0.556; 95% CI, 0.330–0.937; *p* = 0.035, Fig. 2B).

**Table 2:** Investigator-assessed best overall tumor response

Group	No. (%)					
	Total	CR	PR	SD	PD	ORR
Anti-PD-1monotherapy	93 (100.0)	0 (0.0)	29 (31.2)	52 (55.9)	12 (12.9)	29 (31.2)
*Anti-PD-1 plus radiotherapy*						
All sites evaluation	59 (100.0)	2 (3.4)	28 (47.5)	25 (42.4)	4 (6.8)	30 (50.8)
Out-of-field evaluation	46 (100.0)	0 (0.0)	19 (41.3)	23 (50.0)	4 (8.7)	19 (41.3)

*Abbreviations* *CR* complete response, *PR* partial response, *SD* stable disease, *PD* progressive disease, *ORR* objective response rate

**Fig. 3:**
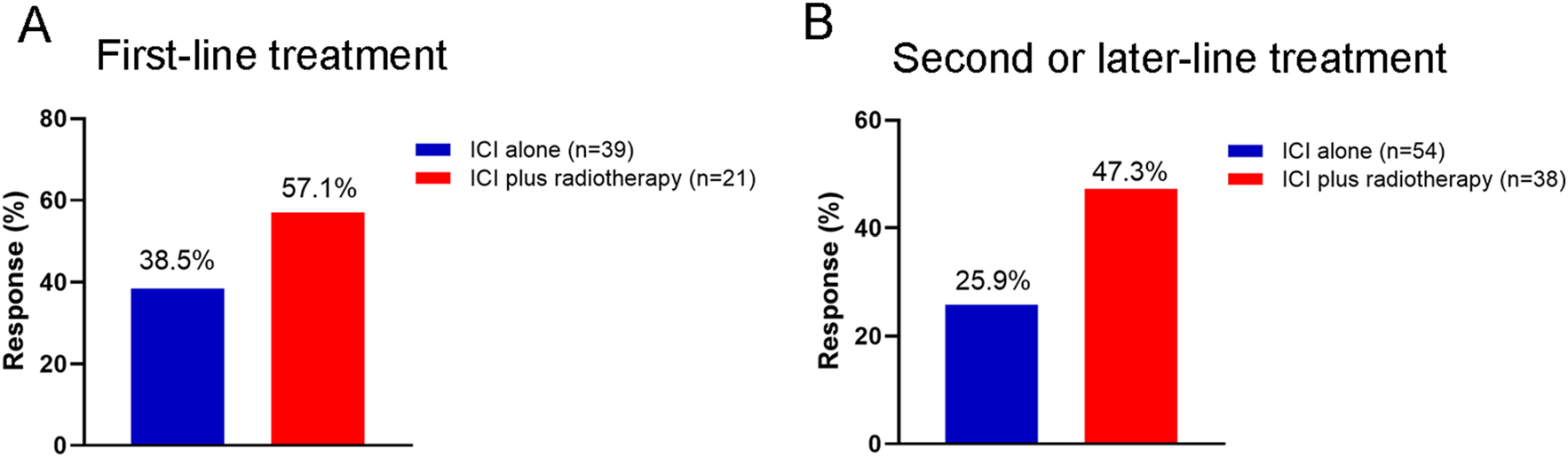
Comparison of best objective response rate (ORR) between the ICI alone group and the ICI plus radiotherapy group in first-line setting (**A**) and second or later-line setting (**B**). *ICI *Immune checkpoint inhibitor

Among the 60 patients who have received PD-1 inhibitors as first-line treatment, the ORR observed were 38.5% in the anti-PD-1 alone group (*n* = 39) and 57.1% in the anti-PD-1 plus RT group (*n* = 21) (Fig. 3A). In the 92 patients receiving PD-1 inhibitors as second- or later-line treatment, the ORRs observed were 25.9% in the anti-PD-1 alone group (*n* = 54) and 47.3% in the anti-PD-1 plus RT group (*n* = 38) (Fig. 3B).

### Efficacy by primary tumor and brain metastases

**Fig. 4:**
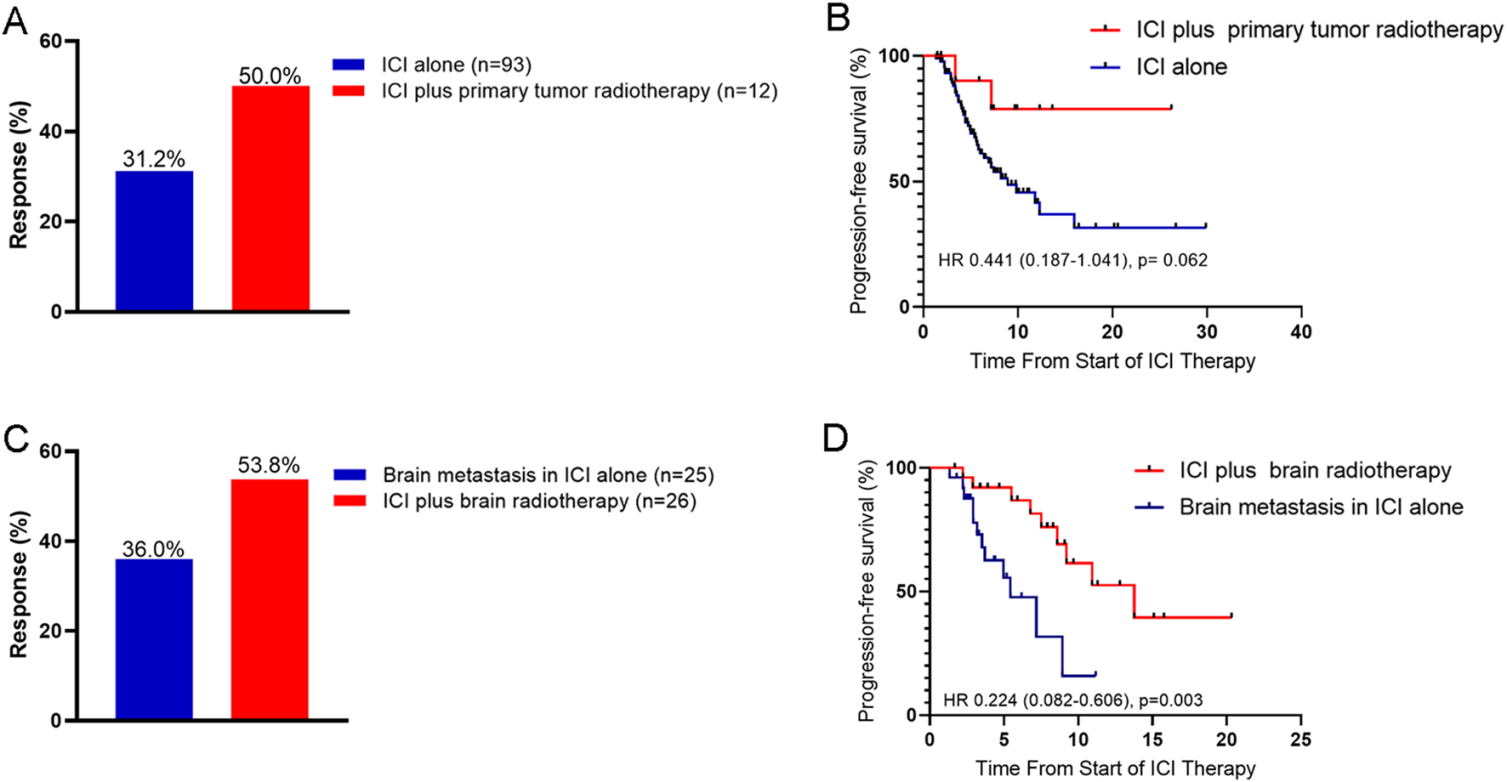
Objective response rate (ORR) and progression-free survival (PFS) categories by primary tumor RT and brain RT (**A**) Best ORR in patients received ICI alone vs. primary tumor RT plus ICI. **B** Kaplan-Meier estimates of PFS in patients for the ICI alone vs. primary tumor RT plus ICI treatment comparison.  **C** Best ORR in patients with brain metastasis received ICI alone *vs.* RT plus ICI. **D** Kaplan–Meier estimates of PFS in patients with brain metastases for the ICI alone versus brain RT plus ICI treatment comparison. *ICI *Immune checkpoint inhibitor

Of the 59 patients in the ICI plus RT group, 12 had primary tumor RT, and 26 had brain RT. Compared with patients in the ICI alone group, primary tumor RT showed an improved ORR (31.2% vs. 50.0%; OR, 0.453, 95% CI 0.14–1.53; *p* = 0.453 Fig. 4A) and a better PFS (HR, 0.441, 95% CI 0.187–1.041; *p* = 0.062 Fig. 4B). In the patients with brain metastasis, brain RT also had a better ORR (36.0% vs. 53.8%; OR, 0.200, 95% CI 0.16–1.48; *p* = 0.200 Fig. 4C) and PFS (HR, 0.224, 95% CI 0.082–0.606; *p* = 0.003 Fig. 4D) compared with brain metastases in ICI alone group.

### Subgroup analysis

**Fig. 5:**
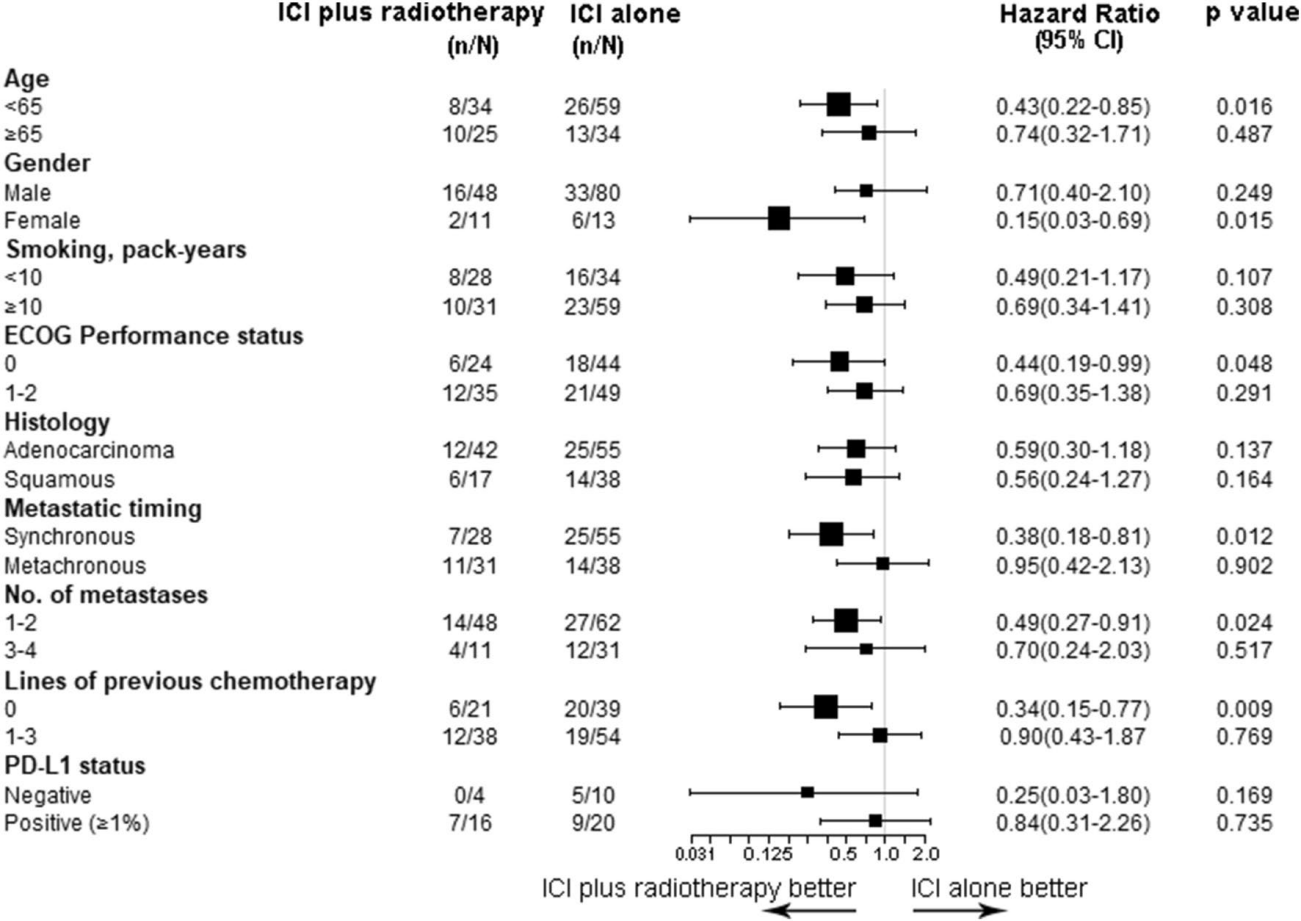
Forest plot of subgroup analysis on progression-free survival

In the subgroup analysis, the combination of ICI plus RT seemed most beneficial among patients aged < 65 years (*p* = 0.016), female patients (*p* = 0.015), ECOG PS = 0 patients (*p* = 0.048), synchronous metastases patients (*p* = 0.012), patients with 1–2 metastatic sites (*p* = 0.024), and patients who received PD-1 inhibitor as first-line therapy (*p* = 0.009) (Fig. 5). A trend toward greater clinical benefit from the addition of RT was seen in the PD-L1-negative subgroup vs. the PD-L1-positive subgroup (Fig. 5).

We performed an exploratory analysis to determine whether any feature was associated with PFS in the ICI plus RT group. As shown in Supplementary Table 2, we could not identify any clinical variables that were significantly associated with PFS in the univariate analysis; therefore, we did not perform multivariable analyses.

### Safety

We conducted a safety evaluation of the ICI plus RT group (Table 3). The overall incidence of AEs was 97% (57 of 59), and most of the observed AEs were grade 1–2 (Table 3). Grade 3–5 treatment-related AEs occurred in nine patients (15%), and one patient died of severe pneumonia. These grade 3–4 AEs were pneumonia (four patients, 7%), bone marrow suppression (four patients, 7%), transaminitis (one patient, 2%), and headache and/or dizziness (one patient, 2%). Most AEs were clinically manageable, with no new toxicity signals.

**Table 3:** Treatment-related adverse events with at least 10% incidence in study population

	No. (%) of Patients (*n* = 59)		
	All grades	Grades 1–2	Grades 3–5
Any adverse event	57 (97)	48 (81)	9 (15)
Fatigue	32 (54)	32 (54)	0
Pain	20 (34)	20 (34)	0
Gastrointestinal response	20 (34)	20 (34)	0
Headache/Dizziness	17 (29)	16 (27)	1 (2)
Bone marrow suppression	12 (20)	8 (14)	4 (7)
Nausea	11 (19)	11 (19)	0
Pneumonia	11 (19)	7 (12)	4 (7)
Transaminitis	9 (15)	8 (14)	1 (2)
Cough	8 (14)	8 (14)	0
Dyspnea	6 (10)	6 (10)	0

## Discussion

This study reported the efficacy and safety of combining single-site RT and ICIs in patients with oligometastatic NSCLC. Our study showed that the addition of single-site RT to immunotherapy could improve ORR and PFS with acceptable AEs. Of note, this combination therapy enhances the occurrence of out-of-field (abscopal) response. The favorable clinical outcomes were also observed for patients with brain metastases. Subgroup analysis revealed that younger patients, patients with a better physical constitution, patients with fewer metastatic sites, and patients who received ICIs as first-line therapy benefited more from the combined approach.

Although no matched paired analysis was performed due to the relatively limited sample size, strict inclusion and exclusion criteria were followed to avoid potential bias. Our results showed that the clinical features were well balanced between the two groups. To further confirm this conclusion, we also performed a subgroup analysis (including brain metastases and primary tumors irradiation). Therefore, we believe our conclusion is interesting enough to warrant large-scale studies.

Oligometastasis with a small disease burden can be classified as an indolent state between the extensive and locally advanced stages. Despite having a relatively short follow-up time for oligometastatic NSCLC patients in the study, anti-PD-1 monotherapy achieved a median PFS of 8.9 months, with an ORR of 31.2%. This was significantly better than the results of the CheckMate 057 and KEYNOTE-001 studies in a second-line setting and was also higher than the median PFS of 6.4 months (KEYNOTE-407) in a first-line setting (Borghaei et al. 2021; Herbst et al. 2016; Paz-Ares et al. 2020). This result suggests that a smaller tumor burden might be necessary for increasing the response to immunotherapy. Local therapy for oligometastatic NSCLC has been shown to improve clinical outcomes in multiple clinical trials (Gomez et al. 2016; Qiu et al. 2017; Weickhardt et al. 2012). RT as a primary local treatment provides local control of the irradiated lesion, and when administered in combination with immunotherapy, enhances antitumor response far outside of the radiation, which is known as the abscopal effect. This phenomenon crucially determines the anti-tumor efficiency of the local RT and ICI combination strategy (Ngwa et al. 2018). However, current strategies designed to test the efficacy of the combination strategy cannot optimally achieve abscopal effects through single-site irradiation in metastatic tumors (Kwon et al. 2014; McBride et al. 2021). In our study, we observed a significant response rate of 50.8% and an out-of-field response rate of 41.3%, which was higher than the ICI alone group (31.2%). Such a trial design is selected for oligometastatic patients with a small disease burden and equally immunogenic tumors that may fully activate the patient’s immune system. In our subgroup analysis, patients with 1–2 vs. 3–4 metastatic sites benefited more from anti-PD-1 treatment plus RT, which further supports the view of a small disease burden in favor of immune responses. In contrast to oligometastatic disease, the heterogeneity of polymetastases means that tumor-associated antigens exposed to RT might not be present at other unirradiated locations, or, if they are present, they might only be recognized in subgroups of the tumor lesion and not in the entire cellular population, making immune clearance at these other unirradiated locations impossible or greatly limited (Easwaran et al. 2014; Heppner and Shekhar 2014; Sharabi et al. 2015; Spiotto et al. 2016).

Undeniably, more biological and clinical evidence supports the use of comprehensive RT delivered to multiple lesions combined with immunotherapy. Irradiating multiple sites helps to increase the likelihood of exposure to both shared and exclusive tumor-associated antigens and promptly reduce tumor burden (Brooks and Chang 2019). A randomized clinical trial from MADCC assessing the effect of combining pembrolizumab with stereotactic body RT showed an out-of-field response rate of 38%, which was much higher than that of PD-1 monotherapy (Welsh et al. 2020). Overall, using multisite irradiation combined with immunotherapy could be beneficial to achieve better therapeutic outcomes. However, this approach is not being widely tested in clinical trials, most likely owing to the lack of official guidelines or fear of AEs.

RT is a local treatment that acts on both the tumor and the surrounding non-malignant tissues; therefore, the likelihood of a successful immunogenic event is also influenced by the tumor microenvironment, the surrounding tissue or organ, and the nodal characteristics of the irradiated site. For example, irradiation of liver metastases in NSCLC patients has resulted in stronger activation of antitumor immunity than the irradiation of pulmonary metastases (Tang et al. 2017). For liver metastases, our sample size is inadequate for accurate analysis. Additionally, the results of our subgroup analyses showed that patients who derived benefit had good prognostic factors, including young age, good body condition, and have received immunotherapy as first-line treatment. Certainly, these optimizations of the ICI combination with RT still need to be validated in prospective clinical trials.

Nevertheless, our study had several limitations. First, this was a retrospective, single-institution analysis with a small sample size, which may introduce selection bias. For example, the percentage of patients with brain metastases in this study was higher than that reported in other studies. Another weakness of our study is the heterogeneity of treatment, including administering different PD-1 pathway inhibitors and different RT regimens; this represents a significant confounding factor. Third, because of the relatively short follow-up period, the survival analysis is limited to PFS, and the conclusion should be interpreted with caution. Moreover, the PD-L1 status of most patients in our study was unknown, making comprehensive subgroup analysis difficult. Nonetheless, we still analyzed the available data, despite being limited.

## Conclusion

In conclusion, our results support that combining single-site RT with PD-1 inhibitors increased responses significantly and improved clinical outcomes in oligometastatic NSCLC patients. This treatment approach warrants further prospective investigation in a randomized clinical trial.

## Supplementary Information

Below is the link to the electronic supplementary material.

Supplementary file1 (DOCX 16 KB)

Supplementary file1 (DOCX 18 KB)

## Data Availability

The datasets used in this study are available from the corresponding author on reasonable request.
