# Supplementary material for: Efficacy of single-site radiotherapy plus PD-1 inhibitors vs PD-1 inhibitors for oligometastatic non-small cell lung cancer
Source: J Cancer Res Clin Oncol. 2021 Nov 23;148(5):1253–61. doi: 10.1007/s00432-021-03849-3 (PMC9015982; doi:10.1007/s00432-021-03849-3)
Supplement: Supplementary file 1 — Supplementary file1 (DOCX 16 KB) [file 432_2021_3849_MOESM1_ESM.docx]

**Table E1.** Dosage regimen of PD-1 inhibitors

| Drug | [Patient](javascript:;)s No. | Doses |
| --- | --- | --- |
| Sintilimab | 57 | 200mg,q3w, intravenously |
| Pembrolizumab | 44 | 200mg,q3w, intravenously |
| Camrelizumab | 22 | 200mg,q2w, intravenously |
| Toripalimab | 15 | 240mg,q2w, intravenously |
| Nivolumab | 14 | 240mg,q2w, intravenously |

Abbreviations:q2w, administered every two weeks;q3w, administered every three weeks.
