# Supplementary material for: Efficacy of single-site radiotherapy plus PD-1 inhibitors vs PD-1 inhibitors for oligometastatic non-small cell lung cancer
Source: J Cancer Res Clin Oncol. 2021 Nov 23;148(5):1253–61. doi: 10.1007/s00432-021-03849-3 (PMC9015982; doi:10.1007/s00432-021-03849-3)
Supplement: Supplementary file 2 — Supplementary file1 (DOCX 18 KB) [file 432_2021_3849_MOESM2_ESM.docx]

**Table 2. Univariate Cox Regression Analysis of progression-free survival( PFS) in** **the anti-PD-1 plus RT group**

| Characteristics | No. of Patients | | | Univariate analysis | | |
| --- | --- | --- | --- | --- | --- | --- |
|  |  |  | HR | 95%CI | p value |  |
| Age |  |  |  |  |  |  |
| <65 | 34 |  | 0.55 | 0.21-1.40 | 0.208 |  |
| ≥65 | 25 |  | 1[Reference] |  |  |  |
| Gender |  |  |  |  |  |  |
| Male | 48 |  | 2.17 | 0.50-9.47 | 0.304 |  |
| Female | 11 |  | 1[Reference] |  |  |  |
| Smoking, pack-years |  |  |  |  |  |  |
| <10 | 28 |  | 0.76 | 0.29-1.98 | 0.579 |  |
| ≥10 | 31 |  | 1[Reference] |  |  |  |
| ECOG Performance status |  |  |  |  |  |  |
| 0 | 24 |  | 0.53 | 0.20-1.43 | 0.212 |  |
| 1-2 | 35 |  | 1[Reference] |  |  |  |
| Histology |  |  |  |  |  |  |
| Adenocarcinoma | 42 |  | 0.88 | 0.33-2.35 | 0.797 |  |
| Squamous | 17 |  | 1[Reference] |  |  |  |
| Primary tumor surgery |  |  |  |  |  |  |
| Yes | 15 |  | 1.01 | 0.37-2.77 | 0.980 |  |
| No | 44 |  | 1[Reference] |  |  |  |
| Metastatic timing |  |  |  |  |  |  |
| Synchronous | 28 |  | 0.68 | 0.26-1.77 | 0.429 |  |
| Metachronous | 31 |  | 1[Reference] |  |  |  |
| Number of metastases |  |  |  |  |  |  |
| 1 | 28 |  | 0.28 | 0.18-1.29 | 0.145 |  |
| 2-4 | 31 |  | 1[Reference] |  |  |  |
| Lines of previous chemotherapy |  |  |  |  |  |  |
| 0 | 21 |  | 0.71 | 0.27-1.90 | 0.495 |  |
| 1-3 | 38 |  | 1[Reference] |  |  |  |
| Systemic treatment options |  |  |  |  |  |  |
| Anti-PD-1 monotherapy | 24 |  | 2.48 | 0.96-6.43 | 0.061 |  |
| Anti-PD-1 and chemotherapy | 35 |  | 1[Reference] |  |  |  |

Abbreviations: ECOG, Eastern Cooperative Oncology Group ; PD-L1, programmed death ligand 1; HR= hazard ratio.
